# Lifestyles and health-related quality of life in Chinese people: a national family study

**DOI:** 10.1186/s12889-022-14680-x

**Published:** 2022-11-29

**Authors:** Shiqian Zou, Guanrui Feng, Danyang Li, Pu Ge, Siyi Wang, Tinlun Liu, Haijun Li, Yongjie Lai, Zijian Tan, Yuling Huang, Jian Huang, Casper Zhang, Yibo Wu, Wai-kit Ming

**Affiliations:** 1 Department of Public Health and Preventive Medicine, School of Medicine, Jinan University, 601 Huangpu W Ave, Tianhe District, Guangzhou, 510632 Guangdong Province China; 2 Medical College of Xi’an Peihua University, Xi’an, China; 3 Key Research Base of Philosophy and Social Sciences in Shaanxi Province, Health Culture Research Center of Shaanxi, Xi’an, China; 4 School of Pharmacy, Jiangxi University of Traditional Chinese Medicine, Nanchang, China; 5 School of Pharmaceutical Science, Shandong University, Jinan, Shandong China; 6 MRC Centre for Environment and Health, Department of Epidemiology and Biostatistics, School of Public Health, St Mary’s Campus, Imperial College London, Norfolk Place, British, London, UK; 7 School of Public Health, University of Hong Kong, Hong Kong, China; 8 Peking University School of Public Health, 38 Xueyuan Road, Haidian District, Beijing, China

**Keywords:** EQ-5D-5L, Health-related quality of life, China, Chinese population, Lifestyles

## Abstract

**Background:**

There were few studies that investigated health-related quality of life (HRQoL) of the general population in China, and many of them reported limitations in sampling.

**Objective:**

To investigate the relationship between lifestyles and HRQoL in the Chinese population in both individual and family levels.

**Method:**

Online questionnaires were distributed across China to collect demographic information and participants’ HRQoL using EuroQoL 5 Dimension scales. The EuroQoL Group’s 5 Dimension scale (EQ-5D) index and EuroQoL Group’s visual analog scale (EQ VAS) score were calculated to evaluate the HRQoL.

**Results:**

A total of 1305 valid questionnaires were included. Higher HRQoL was found in people with intend to lower oil intake, intend to lower salt intake, intend to lower sugar intake, balanced diet, moderate sports every week, a sport hobby and joining a fitness organization (all *p*<.05). HRQoL was higher among male (female as reference), healthy weight (unhealthy weight as reference) (both *p*<.05). Negative correlation was found between HRQoL and clinical medical history and drinking history. Small families (1-2 persons, 83.19 ± 20.14) had poorer HRQoL (EQ VAS score) than big families (≥3 persons, 85.00 ± 17.96, *p* <.05).

**Conclusion:**

In China, people with healthy dietary habits, regular sports habits, healthy weight and male groups tended to have better HRQoL. Clinical medical history and drinking history were negatively related to HRQoL. Small families tend to have poorer HRQoL than big families. The finding implicated influence of the number of family members on people’s perception of health and provided scientific evidence for the current policies to encourage birth in China. For a better HRQoL, we suggest people live in big families and take measures to lower salt/sugar/oil intake and exercise regularly in daily life.

**Supplementary Information:**

The online version contains supplementary material available at 10.1186/s12889-022-14680-x.

## Introduction

Health-Related Quality of Life (HRQoL) is a multi-dimensional indicator for measuring people’s physical, mental, emotional, and social health state in their lives over time. HRQoL not only benefits the health perception at individual level, but also benefits health agencies in legislation, community health plan and business health project [1].

The investigation of HRQoL in China is necessary. The current Chinese healthcare system is overloaded. Compared to other countries, the physician ratio (the number of physician per 10,000 people) in China is not high enough (18.1), which is 1/4 of Cuba (75.2), 1/2 of Norway (43.9) and 3/4 of the United States (25.7) [2]. Moreover, the rapidly aging society is expanding the gap in medical resource deficiency [3]. To relieve the burden on healthcare system, the Chinese government has been advocating healthy lifestyles and taking measures to improve food safety and environmental protection [4, 5]. To achieve this, it is important to explore the potential lifestyle factors related to people’s HRQoL and widely spread the self-care knowledges to everyone in China, which will decrease the risk of many chronic diseases and reduce the national medical burden in China in the future [6].There are many tools available to assess health status, including EuroQoL 5 Dimension (EQ-5D), Assessment of Quality of Life, the Short Form 6D, World Health Organization Quality of Life [7–11]. These scales explore the current health status of the population by compiling data based on participants’ responses to the questions in questionnaires. QoL is a broad multidimensional concept that usually included subjective evaluations of both positive and negative aspects of life, whereas HRQoL is one of many outcomes that are reported by patients, and is mainly about the effects of illness and specifically on the impact of treatment [12]. The construct of HRQoL enables health agencies to legitimately address broader areas of healthy partners, including social service agencies, community planners, and business groups, and is also an important component of public health surveillance [12]. Using HRQoL can not only let us understand the health status of Chinese people, but also provide scientific suggestion for the future public health reform in China. Among them, EQ-5D is an efficiently and commonly used scale to measure people’s HRQoL across the world, especially in China [13–15]. The Short Form 6D questionnaire is a multi-attribute utility instrument to calculate quality-adjusted life years after health care interventions [16]. And this study didn’t plan intervention. Therefore, the Short Form 6D is not a good choice for this study. The World Organization Quality of Life is defined as an individual’s perception of their position in life in the context of the culture and value systems in which they live and in relation to their goals, expectations, standards and concerns [17]. However, it’s hardly used in national health survey in the past. In the past, a lot of studies [18–20] have reported the positive and negative influence of some lifestyle factors on people’s physical health, but few of them [18] have investigated the relationship between lifestyle factors and the people’s HRQoL, which is a comprehensive evaluation of both physical health and mental health. However, prior studies have been conducted to explore the HRQoL among Chinese people, but they highlighted their limitations in sampling [21–23]. A systemic sampling is crucial for the representativeness of the sample to the population. Therefore, it is necessary to establish a study that investigates the HRQoL of Chinese people in a wider range of areas. Apart from that, Chinese families have been sharing the traditional culture of maintaining harmony within families for thousand years, thus people in China are expected to suppress their own interests to achieve the harmony and order in families. Due to the deeply aging structure in China, people might ascribe the change of lifestyle status to the age structure. However, if we use the current lifestyle change of Chinese families, which included all age groups, it can, to a large extent, exclude the impact of age structure. To give public health suggestions for China’s future health policy plan, it is important to understand the quality of life on family basis.

This study aims to explore the relationship between HRQoL and lifestyles of Chinese people and compare the HRQoL among Chinese families.

## Materials and methods

### Study design

A cross-sectional study was performed to study the HRQoL of the general Chinese population using a self-administrated questionnaire, which includes demographic information, lifestyles, and the EuroQol 5 Dimension 5 Level (EQ-5D-5L) scale [24].

The lifestyle questionnaire is pre-validated, the process and statistical result is in additional file (see additional tables). Ethical approval was granted by the Key Research Base of Philosophy and Social Sciences in Shaanxi Province and Health Culture Research Center of Shaanxi (JKWH-2020-21). All participants signed the informed consent documents before participation in this study. Questionnaires were distributed online between October 21^th^, 2020 and October 31^th^, 2020.

### Study population

The questionnaires were designed by the support of the Shaanxi Provincial Key Research Base for Philosophy and Social Sciences and released through the Wenjuanxing platform.

Sampling process in the study was conducted in three stages. First, all participants were extracted using stratified random sampling. Mainland China is composed of seven major administrative regions, and we used random number table to randomly selected two provinces out of each major administrative regions: East China: Shandong and Jiangsu, South China: Guangdong and Hainan, North China: Beijing and Shanxi, Central China: Henan and Hunan, Southwest China: Sichuan and Chongqing, Northeast China: Liaoning and Heilongjiang, and Northwest China: Shaanxi and Xinjiang. In total, 14 regions were decided. Second, since different administrative regions have different population structure characteristics, we designed the sample size, age structure and urban-rural resident ratio proportionate to each of the administrative region population structure. Quota sampling was used in this stage. Finally, we used the snowball sampling method to collect family data. All the participants were asked to invite their first-degree relatives to finish the questionnaire. Participants in the same family had a unique family number, which they filled in the questionnaire for identification of the same family. All investigators were trained to collect questionnaires.

### Measurement

The EQ-5D-5L instrument evaluates individual’s HRQoL through five dimensions (including mobility, self-care, usual activities, pain/discomfort and anxiety/depression). Each dimension has five response levels: no problems, slight problems, moderate problems, severe problems, unable to/extreme problems. And the five response levels were converted in to 1, 2, 3, 4, 5 in the given order. Then a 5L health state vector (e.g. 11111) was obtained. Next, a single EQ-5D index value was calculated through the EQ-5D-5L Crosstalk Index Value Calculator to produce the EQ-5D value, which ranges from -0.111 to 1. 1 refers to the best HRQoL of a person and 0 indicates death, while values less than 0 represent health states regarded as worse than a state that is as bad as being dead [25, 26]. To save space, the official file explaining the EQ-5D-5L Crosswalk_model and methodology had been put into the additional file (see additional figures). The EuroQoL Group’s visual analog scale (EQ VAS) questionnaire was administered in a one-on-one interview. The interviewer showed the subjects a scale ranging from “worst possible (0 points)” to “best possible (100 points)” health, and asked subjects to judge their own health condition comprehensively [27]. Generally, both higher EQ-5D index and higher EQ VAS score indicate better HRQoL.

### Variables

There are some basic independent variates in this study: age, sex, height (cm), weight (kg), Body Mass Index (BMI) (kg/m^2^), educational background, employment status (whether they have job now). Among them, BMI was classified into healthy weight group (18.5-24.9 kg/m^2^) and unhealthy weight (BMI <18.5 or >24.9 kg/m^2^, including underweight (<18.5), overweight (25.0-29.9), and obesity (30.0 and above)) group [28]. Participants’ educational background was divided into 3 categories: no education, low-level education (primary school, middle school and high school), and high-level education (junior college, bachelor degree, master degree and doctorate) [29]. Furthermore, participant’s lifestyles were categorial variables and were included in the study: Oil intake (having taken action to reduce oil intake or no action), salt intake (having taken action to reduce salt intake or no action), sugar intake (having taken action to reduce sugar intake or no action), balanced diet (having taken action to balance diet or no action), moderate exercise per week (having taken action to exercise 2.5 hours per week or no action), having a sport hobby (having has a sport hobby or no action), joining a fitness organization (having joined a fitness organization or no action), smoking history (have smoking habits or no) and drinking history (have drinking habits or no).

### Statistical analysis

Data analysis was performed using the SPSS 24.0 for Windows. Continuous variables were described using the means ± standard deviations (SDs). Categorical variables were described using counts and percentages. The dependent variables were the EQ-5D index and EQ VAS score. Participants’ demographic information were reported. The HRQoL was evaluated by EQ-5D index and EQ VAS score. Subgroup analysis of the EQ-5D index and EQ VAS score was performed for basic demographic informatics. Respondents were classified into two groups (participants with and without disease history) according to whether they had disease history. According to the demographic information, participants were further classified as dichotomous (e.g., male and female), categorical (have smoking habit, have drinking habit), ranked categorical (age: ≤ 35 years old, ≥ 36 or ≤ 55 years old, ≥ 56 years old; educational background: none, low education level (primary school to high school), high education level (junior college to PhD)) or continuous (e.g., weight, height, BMI) [30]. Differences in basic demographic informatics between subgroups were observed by t-test. Following that, all lifestyles are categorial variables that were divided into YES group (having started this lifestyle) and NO group (haven’t started this lifestyle). The EQ-5D indices and EQ VAS scores of the participants with or without these lifestyles (reference group) were compared through t- test to reveal lifestyles’ influence on the HRQoL of participants. Analysis of variance (ANOVA) was used to observe whether the differences in the EQ-5D index and EQ VAS scores due to differences in number of family members were statistically significant, and t-tests were used to observe whether family size also made a difference in the EQ-5D index and EQ VAS scores in order to analyze the effect from the family. And multiple linear regression analysis was run to analyze the correlation between HRQoL and lifestyle factors. All tests were two-sided, and while P value less than 0.05 was considered statistically significant. The average EQ-5D index and EQ VAS score of each province/autonomous region/municipality were shown in the map of China using the Tableau 2020.4 software.

## Results

### Descriptive data

#### Demographic characteristics of participants

In total, 1305 participants from 431 families were included (Table 1) (Table 1 in Tables). For the clinical medical history group, people in which had one or more chronic diseases or complications, 50.16% of them were male and the other 48.84% are female. In this group, male were significantly more than the no clinical medical history group that having no chronic disease or complication (*p* <.05) and female were significantly lesser than the no clinical medical history group (*p* <.05). 22.83% of the clinical medical history group were ≤ 35 years old, and the figures for ≥ 36 or ≤ 55 years old and > 55 years old people were 38.59% and 23.08%, respectively. Significant difference was found in ≤ 35 years old (p <.05) and ≥ 36 or ≤ 55 years old groups (*p* <.05) between those with clinical medical history and without medical history.

**Table 1:** Demographics of respondents

	All respondents (*n*=1305)	Clinical medical history (*n*=311)	No clinical medical history (*n*=994)	*P*-value
Sex				
Male	564(43.22%)	156(50.16%)	408(41.05%)	<.001*
Female	741(56.78%)	155(48.84%)	586(58.95%)	0.009*
Age (years old)				
≤35	716(54.87%)	71(22.83%)	645(49.43%)	<.001*
≥36 or ≤55	409(31.34%)	120(38.59%)	289(22.15%)	<.001*
≥56	180(13.79%)	120(38.59%)	60(4.60%)	0.271
Anthropometric measurements				
Height (*SD*, cm)	163.38(20.62)	162.52(27.06)	163.65(18.19)	<.001*
Weight (*SD*, kg)	69.74(25.78)	72.37(26.68)	68.93(25.45)	<.001*
BMI (*SD*, kg/m^2^)	29.564(32.24)	32.09(34.97)	28.78(31.33)	<.001*
Socio-demographics				
Smoking history	260(19.92%)	101(32.48%)	159(16.00%)	0.192
Drinking history	568(43.52%)	157(50.48%)	411(41.35%)	0.002*
Employment status	1131(86.67%)	202(64.95%)	929(93.46%)	<.001*
Educational background				
No	45(3.45%)	32(10.29%)	13(1.31%)	0.942
Low-level	454(34.79%)	142(45.66%)	312(31.39%)	<.001*
High-level	806(61.76%)	137(44.05%)	669(67.30%)	0.040*

Data with SD indicates “mean” value. Data without SD indicates “count”.

*p< 0.05

The mean values for their average height, weight, BMI were 162.52±27.06 cm, 72.37±26.68 kg, and 32.09±34.97 kg/m^2^, while the figures for participants without clinical medical history were 163.65±18.19 cm, 68.93±25.45kg, and 28.78±31.33 kg/m^2^. The average height in the clinical medical history group was significantly lower than the no clinical history group (*p* <.05).

Table 1 showed the impact of socio-demographic factors on participants. Among them, significant differences were found in drinking history (*p* <.05), employment status (*p* <.05), low-level education (*p* <.05) and high-level education (*p* <.05) between clinical medical history group and no clinical medical history group.

### Outcome data

#### The EQ-5D indices and EQ VAS scores of dietary lifestyles

For the dietary habits, significant higher EQ-5D indices and EQ VAS scores were found in intend to lower oil intake group (n=877, EQ-5D index: 0.88±0.17, *p* <.05; EQ VAS score: 87.00±15.58, *p* <.05), intend to lower salt intake group (n=890, EQ5D index: 0.88±0.16, *p* <.05; EQ VAS score: 86.77±17.13, *p* <.05), intend to lower sugar intake group (*n*=869, EQ-5D index: 0.88±0.16, *p* <.05; EQ VAS score: 86.95±16.42, *p* <.05) and balanced diet (*n*=958, EQ-5D index: 0.88±0.16, *p* <.05; EQ VAS score: 86.73±16.48, *p* <.05) compared to the reference groups (Table 2 in Tables).

**Table 2:** The values of EQ-5D and EQ-5D VAS about lifestyle habits

EQ5D Index (*n*=1305)			
	Intend to lower intake n(Mean, SD)	Reference n(Mean, SD)	*P* value
Dietary habits			
Oil intake	877(0.88,0.17)	428(0.79,0.23)	*p*<.05
Salt intake	890(0.88,0.16)	415(0.78,0.24)	*p*<.05
Sugar intake	869(0.88,0.16)	436(0.77,0.88)	*p*<.05
Balance diet	958(0.88,0.16)	347(0.77,0.25)	*p*<.05
Exercise habits			
Moderate exercise (2.5h) every week	783(0.89,0.15)	522(0.78,0.24)	*p*<.05
Having at least one sport hobby	782(0.89,0.15)	523(0.78,0.23)	*p*<.05
Joining a fitness organization	595(0.89,0.16)	710(0.81,0.21)	*p*<.05
Smoking and drinking history			
Smoking history	260(0.82,0.21)	1045(0.85,0.19)	0.139
Drinking history	568(0.82,0.19)	737(0.87,0.20)	0.552
EQ-5D VAS (n=1305)			
Dietary habits			
Oil intake	877(87.00,16.58)	428(78.44,22.07)	*p*<.05
Salt intake	890(86.77,17.13)	415(78.65,21.45)	*p*<.05
Sugar intake	869(86.95,16.42)	436(78.70,22.29)	*p*<.05
Balance diet	958(86.73,16.48)	347(77.18,23.23)	*p*<.05
Exercise habits			
Moderate exercise (2.5h) every week	783(88.20,16.01)	522(78.18,21.38)	*p*<.05
Having at least one sport hobby	782(88.01,16.99)	523(78.48,20.34)	*p*<.05
Joining a fitness organization	595(88.72,16.80)	710(80.39,19.87)	*p*<.05
Smoking and drinking history			
Smoking history	260(81.89,19.75)	1045(84.76,18.76)	0.050
Drinking history	568(81.56,20.47)	737(86.22,17.51)	*p*<.05

#### The EQ-5D indices and EQ VAS scores of exercise habits

Significant higher EQ-5D indices and EQ VAS scores were also revealed in exercise habits (Table 2 in Table), including moderate exercise (2.5h) every week (*n*=783, EQ-5D index: 0.89±0.15, *p* <.05; EQ VAS score: 88.20±16.01, *p* <.05), having at least one sport hobby (*n*=782, EQ-5D index: 0.89±0.15, *p* <.05; EQ VAS score: 88.01±16.99, *p* <.05), and joining a fitness organization (*n*=595, EQ-5D index: 0.89±0.16, *p* <.05; EQ VAS score: 88.72±16.80, *p* <.05), compared to reference group.

The results on the smoking history and drinking history (Table 2 in Tables) showed that only the EQ VAS score on drinking history was significantly different between the Yes group and No group (*n*=568; 56±20.47, *p* <.05).

We also compared the EQ-5D indices and EQ VAS scores among different families with different family sizes. The EQ-5D index showed significantly different EQ-5D index among 1 person families, 2 persons families, 3 persons families and more than 4 persons families (*p* <.05), but no significant result was found for EQ VAS score (Table 3 in Tables).

**Table 3:** Comparison between family groups

Family size	EQ-5D index *n* (*Mean, SD*)	EQ VAS score *n* (*Mean, SD*)
1 person	35(0.85,0.13)	35(84.46,15.48)
2 persons	552(0.83,0.21)	552(83.11,20.41)
3 persons	156(0.84,0.20)	156(85.07,17.46)
≥4 persons	562(0.87,0.18)	562(84.98,18.11)
*P*-value	0.008*	0.377

*p < 0.05

For the comparison between small families and big families (Table 4 in Tables), significant higher EQ VAS score was found in big families (p <.05).

**Table 4:** comparison between family groups

Family size	EQ-5D index *n* (*Mean, SD*)	EQ VAS score *n* (*Mean, SD*)
Small family (1-2 persons)	587(0.83,0.21)	587(83.19,20.14)
Big family (≥3 persons)	718(0.86,0.18)	718(85.00,17.96)
*P*-value	0.742	0.017*

* *p* < 0.05

**Fig. 1:**
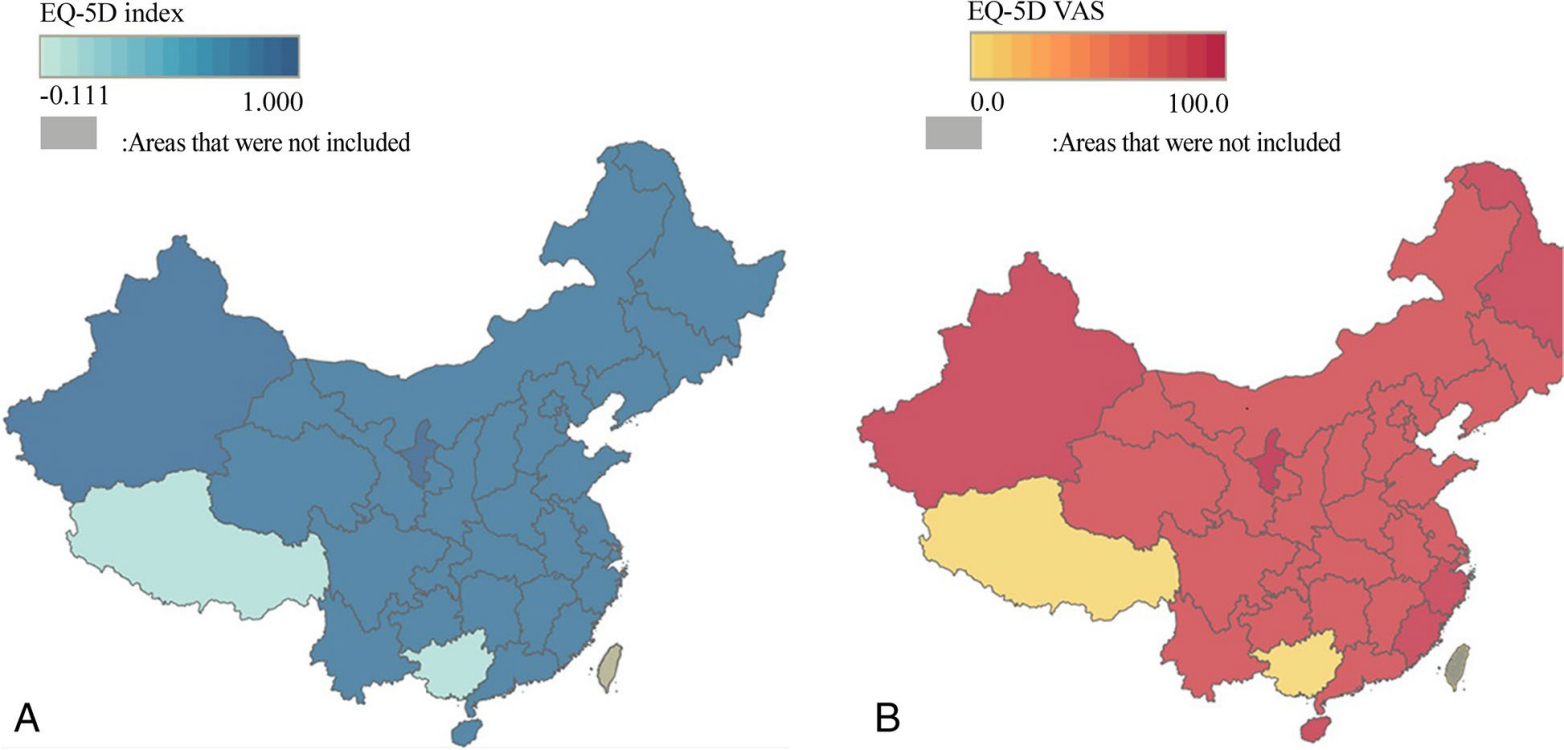
A Cartograms of China with the area of each province in mainland China weighted by sample group. Respondents are shaded according to: **A** the average EQ-5D values of each province; **B** The average EQ VAS score of the respondents in each province

According to the population movement, although we extracted the sample from 14 provinces/municipalities/autonomous regions, the participants in the sample were from places across mainland China. Therefore, we geocoded the mean EQ-5D values for each province in mainland China based on the address on the People's Republic of China resident identity card of the participants. The results about the HRQoL of different regions were in the additional file (see additional tables). According to the EQ-5D index map (Picture A of Fig. 1), respondents in Ningxia and Xinjiang provinces of China had higher average EQ-5D index, while respondents in Tibet and Guangxi provinces had lower average EQ-5D index. For the EQ VAS score map (picture B of Fig. 1), respondents in Ningxia, Xinjiang, Guangdong, Fujian and Hainan provinces were found to have higher average EQ VAS score, and lower average EQ VAS score was found in Tibet and Guangxi provinces.

The correlations were assessed by multiple linear regression (Table 5 in Tables), which revealed that the balanced diet (both *p* <.05), moderate exercise every week (both *p* <.05) were found to be positively correlated to both EQ5D index and EQ VAS score, and clinical medical history (both *p* <.05) and drinking history (both *p* <.05) was found to be negatively correlated with both EQ5D index and EQ VAS score.

**Table 5:** The association between lifestyles and HRQoL, with EQ-5D indices and EQ VAS score as dependent variables, respectively

	Coefficient					
	EQ-5D Index (*R*=0.46, R^2^=0.22)	95%CI	*p*-value	EQ-5D VAS (*R*=0.41, R^2^=0.17)	95%CI	*p*-value
Clinical medical history (no as reference)	-0.14	(-0.16 - -0.12)	*p*<.05	-10.82	(-13.07 - -8.57)	*p*<.05
Gender (female as reference)	-	-	-	2.32	(0.18 – 4.46)	*p*<.05
BMI (unhealthy weight as reference)	-	-	-	2.51	(0.61 – 4.42)	*p*<.05
Intend to lower oil intake (reference group as reference)	-	-	-	2.94	(0.54 – 5.35)	*p*<.05
Intend to lower sugar intake (reference group as reference)	0.05	(0.03 – 0.08)	*p*<.05	-		-
Balanced diet (reference group as reference)	0.05	(0.02 – 0.07)	*p*<.05	4.63	(2.06 – 7.20)	*p*<.05
Moderate exercise every week (reference group as reference)	0.05	(0.02 – 0.07)	*p*<.05	6.76	(4.62 – 8.90)	*p*<.05
Having at least one sport hobby (reference group as reference)	0.03	(0.00 – 0.05)	*p*<.05	-		-
Drinking history (no as reference)	-0.03	(-0.05 - -0.01)	*p*<.05	-4.57	(-6.70 - -2.45)	*p*<.05
F test	57.81		*p*<.05	37.76		*p*<.05

A single “-” in the column indicates that the factor is not included in this multiple linear regression equ

Apart from that, in this multiple linear regression model, although there was a small coefficient of determination r2, the overall significance F-test results for this model still indicated that the dependent variable is linearly correlated with many of the independent variables [31]. There were correlations between EQ-5D index and intend to lower sugar intake (*p* <.05) and having at least one sport hobby (*p* <.05), while no correlation was found between these two lifestyles and EQ VAS score (Table 5 in Tables). On the contrary, there were positive correlation between EQ VAS score and sex (*p* <.05), BMI (*p* <.05), and intend to lower oil intake (*p* <.05), but no correlation was found between them and EQ-5D index (Table 5 in Tables).

## Discussion

### Principal findings

Respondents with some lifestyles (dietary habits: intend to lower oil intake, intend to lower salt intake, intend to lower sugar intake, and balanced diet and sports habits: moderate sport, a sport hobby, joining a fitness organization) had higher EQ-5D index and EQ VAS score, i.e., better HRQoL, compared with other respondents who don’t have the lifestyles. Balanced diet and moderate exercise every week were positively related to EQ-5D index and EQ VAS score. Clinical medical history and drinking history were negatively related to EQ-5D index and EQ VAS score. Intend to lower sugar intake, having at least one sport hobby were positively related to EQ-5D index, while sex (female as reference), BMI (unhealthy weight as reference), and intend to lower oil intake were positively related to EQ VAS score. People in Xizang and Guangxi provinces had lower average HRQoL. Small families (1-2 persons, 83.19 ± 20.14) had poorer HRQoL (EQ VAS score) than big families (≥3 persons, 85.00 ± 17.96, *p* <.05).

### Strengths and weaknesses of the study

This study is the first one that collecting and analyzing data from family unit to assess HRQoL in mainland China. This study collected information from participants across the mainland China, which is, to the date, the widest sampling area in the researches about HRQoL in mainland China. This study is the first one to explore the correlation between HRQoL and dietary habits, sports habits across China.

This study has several limitations. First, we only use EQ-5D-5L questionnaire to assess individual’s HRQoL, but not comparing the effectiveness of other questionnaires. Second, our study revealed the correlation between lifestyle factors and HRQoL, but the causation hasn’t been revealed. Third, this study was conducted during the COVID-19 pandemic, the increased food price and long-term quarantine might affect people’s dietary intake and mental health respectively. Fourth, the questionnaire was only available for 10 days (October 21th to October 31th), which is relatively short and all in autumn. However, study showed that the seasonal changes in mood and behavior are associated with the HRQoL and mental well-being [32].

### Strengths and weaknesses in relation to other studies

This study has shown that higher average HRQoL was found in people with dietary factors (intend to lower oil intake, intend to lower salt intake, intend to lower sugar intake, and balanced diet). The intend to lower oil intake lifestyle was negatively correlated to the HRQoL in Chinese people, which was similar to the previous study in Spain [33]. It had revealed a detrimental relationship between trans saturated fatty acids intake and most of the mental domains (including vitality, social functioning and role emotional) of QoL [ 34]. However, physical diseases will undoubtedly impact people's HRQoL. And there was no such research that systematically investigate the relationship between intend to lower oil intake and HRQoL in China before. The Harvard Health Publishing has reported that consuming too much sugar can raise blood pressure and increase chronic inflammation, both of which were pathological pathways to heart disease, and the impacted physical disease will affect HRQoL [35]. Too much added sugar can be one of the greatest threats to chronic diseases, such as diabetes, heart disease, fatty liver disease, and some cancers. Study has shown that excess dietary sodium not only increase blood pressure, but also increase arterial stiffness, decrease glomerular filtration rate, increase left ventricular hypertrophy, and sensitized sympathetic neurons [36]. As a result, elevated dietary sodium adversely affected target organs, including blood vessels, heart, kidneys and brain [37]. Although the benefits of salt, sugar reduction and balanced diet had been proven and suggested by World Health Organization (including reducing blood pressure, risk of cardiovascular disease, stroke and coronary heart attach), there was no research that ever investigated the correlation between salt, sugar intake and HRQoL [38–40]. The result on balanced diet was consistent to the study in the past, which concluded that balanced diet improves quality of life and provides an adequate quantity of fermentable carbohydrates [41].

Respondents who had sports habits (including having a sport hobby, joining a fitness group and doing moderate exercise every week) had been revealed to have a better HRQoL, which was also consistent to the previous researches [42, 43]. Omorou, Y.A., et al had concluded in a study incorporating Frenches that sport was nearly always associated with better quality of life, even more so for people who has high physical activity levels [42]. Another study indicated that high sports-active showed better scores on almost all dimensions of HRQoL than low-sports active children and non-sports club members, and frequency of sports is more relevant to HRQoL than the form of sports participation [43]. A number of studies reported that HRQoL is reversely related to BMI [ 44–47]. Our study gave a positive correlation between BMI (unhealthy weight as reference) and EQ VAS score, and no correlation to the EQ-5D index. A study on Chinese population revealed that obesity impaired physical but not mental health [48]. This unsignificant result on EQ-5D index may be due to the different impact of BMI on mental health and physical health (EQ-5D-5L questionnaire assesses both physical health and mental health).

We also found that in our study, male tend to have higher HRQoL. Many studies in the past revealed similar results, which reported that the HRQoL scores were poorer in female than male [49]. This might account for the higher average educational background and the higher family pressure of female. Higher education background indicated greater competition, following with a busy life and less free time. At the same time, China had one of Asia-Pacific’s highest labor force participation rates for female, and female made up 43.7% of the total labor force in 2019 [50]. Therefore, female shoulder both work and family tasks (including housework, childbirth, fostering children, etc).

The worse HRQoL in further southwest China might due to the relatively poorer economics status there [51]. Study have reported that a higher QoL is found in wealthier nations [52].

The negative correlation between HRQoL and drinking history is consistent to some previous studies [53, 54]. However, there were studies reporting alcohol drinkers, including those with heavy drinking, reported better physical HRQoL than non-drinkers [55, 56]. This might be reverse causation, in which people with better physical HRQoL can get drunk, and non-drinkers cannot, for example, people who were ≤ 35 years old tend to be alcoholic and people who were > 55 years old tend to avoid alcohol due to decreased physical fitness.

Small families (1-2 persons) had statistically poorer HRQoL compared to big families (≥3 persons). As the sustained high pressure on work and life, many people in China have to delay conception or choose no kids [57]. Therefore, the small family size may be a resultant factor of lower HRQoL.

### The meaning of the study

For the future health policy planning, we suggest that the Chinese government should make reasonable efforts to improve healthy lives of the people, especially in the parts of health education and promotion through evidence-based medicine, such as the health-promoting factors in our results: reducing oil, salt and sugar intake, balanced diet, moderate exercise every week, joining a fitness organization and having at least one sports hobby. The government can consider using television programs to cultivate people's healthy eating habits, building more sports facilities to encourage regular exercise [58]. Investing the early childhood institutions and giving birth allowance are possible ways to alleviate people’s pressure and improve the average HRQoL of the population [59]. All in all, the improvement of HRQoL for the Chinese people requires long-term and continuous efforts by both the government's guidance and the people themselves to response and practice [60]. We believe that this study provides a powerful and scientific evidence of Chinese people’s HRQoL and the related factors and might be helpful to the Chinese government in the health planning of China in the future.

### Unanswered questions and future research

It is still not clear whether these lifestyle factors are causative or resultant to high HRQoL, and the underlying reasons are expected to be revealed in future researches. In the future, studies with larger sample size and different health scales are expected to investigate the impact of drinking on HRQoL.

## Conclusion

In China, people with healthy dietary habits, regular sports habits, lower BMI and male groups tend to have better HRQoL. Clinical medical history and drinking history were negatively related to HRQoL. Small families tend to have poorer HRQoL (EQ VAS score) than big families. The finding implicated influence of the number of family members on people’s perception of health and provided scientific evidence for the current policies to encourage birth in China. The study result is applicable to the whole Chinese population, as all participants were extracted using stratified random sampling, where participants were from the 14 randomly selected provinces/autonomous region/municipality across all 31 administrative regions in mainland China. It is highly representative and inferential.

## Supplementary Information

**Additional file 1.**

**Additional file 2.**

## Data Availability

All data generated or analysed during this study are included in this published article [and its supplementary information files].

## Abbreviations

HRQoL: health-related quality of life
EQ-5D-5L: EuroQol 5 Dimension 5 Level
EQ VAS: EuroQoL Group’s visual analog scale
EQ-5D: EuroQoL Group’s 5 Dimension scale
BMI: Body Mass Index
SD: standard deviation
ANOVA: Analysis of variance
