# Supplementary material for: Lifestyles and health-related quality of life in Chinese people: a national family study
Source: BMC Public Health. 2022 Nov 29;22:2208. doi: 10.1186/s12889-022-14680-x (PMC9706972; doi:10.1186/s12889-022-14680-x)
Supplement: Supplementary file 1 — Additional file 1. [file 12889_2022_14680_MOESM1_ESM.docx]

The lifestyle questionnaire has undergone expert panel discussion. The lifestyle questionnaire had been pre-validated. 14 provinces were decided through stratified random sampling. The pre-experiment used quota sampling to sample 50 persons in each of the 14 provinces. The questionnaires were designed by Key Research Base of Philosophy and Social Sciences in Shaanxi Province and Health Culture Research Center of Shaanxi and released by Wenjuanxing platform. The questionnaires collected in pre-experiment were not included in the final formal research.

This study confirmed the reliability and validity of the questionnaire by SD, CITC, exploratory factor analysis, Cronbach’s α coefficient. All of them (SD > 1, CITC > 0.5, Cronbach’s α > 0.8, factor analysis > 0.6) are standards implicating that the questionnaire has high reliability and validity. The results were showed in table 1.

Table 1. Questionnaire’s reliability and validity analysis.

|  | SD | CITC | Cronbach's Alpha if item deleted(Total=0.851) | Factor analysis |
| --- | --- | --- | --- | --- |
| Dietary habits |  |  |  |  |
| Oil intake | 4.586 | 0.648 | 0.825 | 0.769 |
| Salt intake | 4.535 | 0.673 | 0.821 | 0.790 |
| Sugar intake | 4.612 | 0.632 | 0.827 | 0.752 |
| Balance diet | 4,736 | 0.617 | 0.830 | 0.737 |
| Exercise habits |  |  |  |  |
| Moderate exercise (2.5h) every week | 4.712 | 0.570 | 0.825 | 0.679 |
| Having at least one sport hobby | 4.668 | 0.598 | 0.833 | 0.704 |
| Joining a fitness organization | 4.784 | 0.545 | 0.840 | 0.656 |

Based on Yes group and No group, we analyzed participants’ lifestyle through t test. And we observed that there’s EQ 5D index statistical difference and EQ-5D VAS score statistical difference between people having those lifestyles and people not having those lifestyles.

Table 2.The values of EQ-5D and EQ-5D VAS about lifestyle.

| EQ5D Index (n=750) | | | |
| --- | --- | --- | --- |
|  | Yes group  n(Mean, SD) | No group  n(Mean, SD) | P value |
| Dietary habits |  |  |  |
| Oil intake | 445(0.82,0.20) | 305(0.76,0.27) | <0.05 |
| Salt intake | 438(0.85,0.20) | 312(0.77,0.27) | <0.05 |
| Sugar intake | 440(0.85,0.20) | 310(0.77,0.27) | <0.05 |
| Balance diet | 518(0.86,0.18) | 232(0.71,0.31) | <0.05 |
| Exercise habits |  |  |  |
| Moderate exercise (2.5h) every week | 412(0.86,0.20) | 338(0.77,0.27) | <0.05 |
| Having at least one sport hobby | 430(0.86,0.20) | 320(0.76,0.27) | <0.05 |
| Joining a fitness organization | 299(0.86,0.20) | 451(0.79,0.25) | <0.05 |
| EQ-5D VAS (n=1305) | | | |
| Dietary habits |  |  |  |
| Oil intake | 445(81.13,20.79) | 305(76.52,23.23) | <0.05 |
| Salt intake | 438(83.88,20.90) | 312(77.26,23.24) | <0.05 |
| Sugar intake | 440(84.56,19.88) | 310(76.26,24.19) | <0.05 |
| Balance diet | 518(85.08,19.02) | 232(72.31,25.79) | <0.05 |
| Exercise habits |  |  |  |
| Moderate exercise (2.5h) every week | 412(85.01,19.66) | 338(76.40,24.01) | <0.05 |
| Having at least one sport hobby | 430(85.11,19.60) | 320(75.78,24,16) | <0.05 |
| Joining a fitness organization | 299(85.29,20.81) | 451(78.37,22.57) | <0.05 |

Table 3.The HRQoL of different regions in China.

| Regions | EQ-5D index | EQ-5D VAS score |
| --- | --- | --- |
| Anhui | 0.84612 | 84.131 |
| Beijing | 0.84668 | 84.212 |
| Fujian | 0.8509 | 84.692 |
| Gansu | 0.84474 | 84.232 |
| Guangdong | 0.84824 | 84.262 |
| Guangxi | 0.009 | 5.231 |
| Guizhou | 0.84953 | 83.589 |
| Hainan | 0.85083 | 84.623 |
| Hebei | 0.84688 | 84.275 |
| Henan | 0.84803 | 84.289 |
| Heilongjiang | 0.85004 | 84.693 |
| Hubei | 0.84822 | 84.236 |
| Hunan | 0.84993 | 84.539 |
| Jilin | 0.84907 | 84.559 |
| Jiangsu | 0.8476 | 84.601 |
| Jiangxi | 0.84081 | 83.863 |
| Liaoning | 0.84932 | 84.284 |
| Inner Mongolia | 0.84475 | 84.464 |
| Shandong | 0.84802 | 84.277 |
| Shanxi | 0.84878 | 84.275 |
| Shaanxi | 0.84855 | 84.283 |
| Shanghai | 0.84754 | 84.495 |
| Sichuan | 0.8435 | 84.131 |
| Tianjin | 0.84672 | 84.53 |
| Xinjiang | 0.85746 | 84.916 |
| Yunnan | 0.8381 | 84.092 |
| Zhejiang | 0.85107 | 84.683 |
| Chongqing | 0.8485 | 84.255 |
| Ningxia | 0.007 | 3.732 |
| Tibet | 0.84781 | 84.698 |
| Qinghai | 0.84576 | 84.143 |
