# Supplementary material for: Lifestyles and health-related quality of life in Chinese people: a national family study
Source: BMC Public Health. 2022 Nov 29;22:2208. doi: 10.1186/s12889-022-14680-x (PMC9706972; doi:10.1186/s12889-022-14680-x)
Supplement: Supplementary file 2 — Additional file 2. [file 12889_2022_14680_MOESM2_ESM.pdf]

INTERIM SCORING FOR THE EQ-5D-5L: MAPPING THE EQ-5D-5L TO EQ-5D-3L VALUE SETS

Methodology

The crosswalk was based on a response mapping approach that estimated the relationship between responses to the EQ-5D-3L ('3L') and EQ-5D-5L ('5L') descriptive systems, and subsequently established a link to the 3L value sets.

In this model, 3 responses were predicted from 5L responses. The probabilities associated with the 3L responses were then applied to their index values to obtain 5L values, as follows.

To initially predict 3L responses, simple non-parametric calculations based on the frequencies obtained when cross tabulating the responses on the 3L and 5L were used, i.e. the proportions of the 3L level scores within each of the five 5L levels. This "non-parametric" model leads, for each dimension and level of the 5L, to probabilities of being in each of the 3L levels. For each health state described by the 5L system (n=3125), the probability of reporting each of the 243 3L health states was estimated by taking the product of the corresponding probabilities. For instance, a respondent reporting the 5L health state vector 23245 and the 3L health state vector of 12123 is the product of:

the probability of level 1 on 3L-Mobility given level 2 on 5L-Mobility;
the probability of level 2 on 3L-Self Care given level 3 on 5L-Self Care;
the probability of level 1 on 3L-Usual Activities given level 2 on 5L-Usual Activities;
the probability of level 2 on 3L-Pain/Discomfort given level 4 on 5L-Pain/Discomfort;
the probability of level 3 on Anxiety/Depression given level 5 on 5L-Anxiety/Depression.

In total, 243 transition probabilities for the 3L are generated for each 5L health state. Note that in this model we did not allow for interaction between the dimensions. The 5L index value is then calculated by multiplying the 243 transition probabilities by their corresponding 3L index values, and subsequently summing them. This can be done for each 5L health state linked to each 3L health state. In this way, a 3125 x 243 matrix of transition probabilities was created.

Results

Respondents completed both the 3L and 5L in 6 countries: Denmark, England, Italy, the Netherlands, Poland and Scotland. Different subgroups were targeted, and in most countries a screening protocol was implemented to ensure that a broad spectrum of levels of health would be captured across the dimensions of EQ-5D for both the 5L and 3L descriptive systems.

In total, 3691 respondents completed both the 3L and 5L. The overall cohort was 53% female and had a mean age of 51.5 (standard deviation = 20). Respondents were classified into different subgroups that included COPD/asthma, diabetes, liver disease, rheumatoid arthritis/arthritis, cardiovascular disease, stroke, depression, personality disorders, students, and "other conditions".

[1] M. Herdman et al. Qual Life Res DOI 10.1007/s11136-011-9903

The in-sample prediction (fit) for the final model resulted in a mean square error (MSE) of 0.014. Also, a number of out-of-sample predictions (predictive power) were performed on the condition groups mentioned above that resulted in an MSE range from 0.005 to 0.021.

Figure 1. Data fit for final model

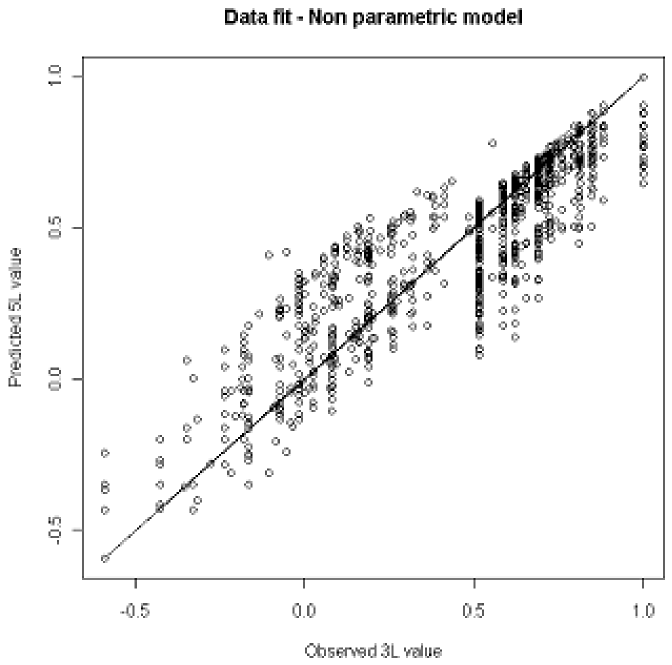

EQ-5D-5L Crosswalk Value sets

If you follow this link you can download an Excel file containing the EQ-5D-5L value sets for the following countries: France, Germany, Japan, the Netherlands, Spain, Thailand, UK, US and Zimbabwe. In the Excel file there are three sheets: (1) 'Transition probabilities' presenting the transition probabilities when going from a 5L level to a 3L level for each dimension; (2) 'Probability matrix' gives the transition probabilities for all 5L to 3L health states; (3) 'EQ-5D-5L value sets' gives the EQ-5D-5L index values based on the crosswalk for the abovementioned countries.

A tool which will allow users to calculate EQ-5D-5L index values for their dimension responses will follow shortly.

Limitations

This study has several limitations, some of which are common to mapping studies. One of the major limitations of any mapping study is the potential difference in content coverage. In this respect, the present study is well-suited to a mapping approach because the dimensions of the EQ-5D-3L and EQ-5D-5L are identical. A limitation of the present study related to mapping is that any mapping is data dependent; therefore the selection of respondents can influence the calibration of values. For this reason, the data collection phase was designed to facilitate a wide range of levels of health across the different dimensions

[1] M. Herdman et al. Qual Life Res DOI 10.1007/s11136-011-9903

on the EQ-5D in a large number of respondents from different counties. The out-of-sample validation indicates that the mean square error of the predictions stay within an acceptable range (< 0.021).

A second limitation relates to restrictions on the range of scale possible for 5L values when mapping to 3L value sets. Specifically, respondents who categorized themselves as 55555 using 5L cannot report a health state worse than 33333 using 3L, yet it is possible for them to report e.g. 23333. For this reason, a crosswalk-based approach does not allow the value of 55555 to be lower than that of 33333. This limitation places an artificial floor effect on the values of 5L while 5L values - when valued directly - might in fact be lower when compared to the 3 level system. A final limitation is that 3L and 5L dimension scores were pooled from various countries, using different translations of the 5L descriptive system. There might have been cultural differences in how respondents from the various countries interpreted the different 5L translations. The only way to deal with this problem would be to develop a crosswalk for each country separately, but this was not deemed feasible. Furthermore, inter-country results from the UK and Spain showed that the 5L labels performed substantially similarly on the response scaling task[1].

[1] M. Herdman et al. Qual Life Res DOI 10.1007/s11136-011-9903
